# The Impact of Citric Acid Solution on Hydraulic Calcium Silicate-Based Sealers and Root Dentin: A Preliminary Assessment

**DOI:** 10.3390/ma17061351

**Published:** 2024-03-15

**Authors:** Saulius Drukteinis, Goda Bilvinaite, Simas Sakirzanovas

**Affiliations:** 1 Institute of Dentistry, Faculty of Medicine, Vilnius University, Zalgirio 115, LT-08217 Vilnius, Lithuania; goda.bilvinaite@mf.stud.vu.lt; 2 Department of Applied Chemistry, Institute of Chemistry, Vilnius University, Naugarduko 24, LT-03225 Vilnius, Lithuania; simas.sakirzanovas@chf.vu.lt

**Keywords:** calcium silicate, sealer, citric acid, EDTA, retreatment, root canal, SEM, solvent

## Abstract

Hydraulic calcium silicate-based (HCS) sealers have recently gained tremendous popularity due to their unique properties. However, their removal during endodontic retreatment is challenging. The solvent, which could chemically deteriorate the material, would be highly desirable for endodontic retreatment procedures. This preliminary study assessed the interplay and dissolving capability of 10% and 20% citric acid, compared to 17% EDTA, on commonly used HCS sealers (AH Plus Bioceramic Sealer, Bio-C Sealer, BioRoot RCS, TotalFill BC Sealer), and evaluated the potential impact of these solutions on root dentin structure. The interaction between tested sealers and irrigating solutions was photographed, and solubility-related mass changes were determined. The surface morphology of treated filling materials and dentin was evaluated using a scanning electron microscope (SEM). One-way analysis of variance (ANOVA) along with Tukey’s test were used to detect the statistically significant differences among groups at the confidence level of 0.95. Intense gas release was observed during the interaction of HCS materials and citric acid, with no evidently visible “bubbling” after the immersion in EDTA. The mass loss of HCS sealers equally confirmed the significantly higher dissolving characteristics of 10% and 20% citric acid solutions compared to EDTA. The surface structural changes, associated with pore and crack formation, were mainly seen for HCS sealers exposed to citric acid. Meanwhile, no severe erosion was detected for dentin after root canal preparation with 10% and 20% citric acid solutions. These findings demonstrate that citric acid has the potential to dissolve HCS sealers with minimal or no negative impact on root dentin, suggesting citric acid as a solvent for HCS sealers in endodontic retreatment procedures.

## 1. Introduction

The success of endodontic treatments is significantly affected by the quality of root canal obturation and the materials used, including endodontic sealers [1]. These materials must ensure a three-dimensional seal within the root canal system, preventing re-infection and promoting the long-term success and survival of the endodontically treated tooth [2]. Despite meticulous efforts during the initial endodontic treatment procedures, the success rates of primary endodontics never reach 100% [3]. The need for endodontic retreatment usually arises if there is a persistent or recurrent infection and the tooth continues to exhibit symptoms, e.g., persistent pain, swelling, or inflammation [4]. Additionally, various problems and complications, such as missed root canals, incomplete removal of the pulp tissue, and non-hermetic root canal sealing, may warrant a second intervention [5].

The ability to completely remove the previous root canal filling material, typically the gutta-percha and the sealer, is crucial for precise root canal disinfection and subsequent re-obturation during endodontic retreatment. Factors such as the type of sealer, its chemical composition, and its interaction with root canal walls highly affect how easily the sealer can be dislodged and removed [6]. However, unlike the gutta-percha, most sealers do not have any specific solvent, facilitating their removal from the root canal system [6,7]. This problem equally applies to all types of hydraulic calcium silicate-based (HCS) materials, which are currently the most popular clinical choice and are highly recommended by various clinical guidelines and evidence-based data [8].

Over the years, various endodontic sealers have been developed, each possessing unique properties, advantages, and disadvantages [9]. The choice of sealer often depends on factors such as the specific clinical scenario, the clinician’s experience and preference, and the desired properties of the material suitable to the particular biological conditions of every clinical case [10]. HCS sealers significantly changed the fundamental principles of root canal obturation, which focused on higher amounts of gutta-percha and less sealer [11]. Due to the lack of shrinkage and long-term dimensional stability, HCS materials overcame the need to increase the gutta-percha/sealer ratio and, thus, were advocated for the simplified obturation concept based on the single tapered gutta-percha point and HCS sealer [12]. This root canal filling technique highly simplified the daily clinical practice, even for operators with limited clinical experience, while equally providing high clinical success rates in primary endodontic treatment and retreatment [13].

However, the retreatment of root canals previously obturated with HCS sealers usually poses a significant challenge. Owing to the adhesion of HCS materials to the dentin via mineral infiltration zone formation and the deeper penetrability into dentinal tubules, these sealers are hardly removed from the root canal system, even mechanically [14]. The previously tested irrigating solutions, such as EDTA, NaOCl, carbonated water, and formic and acetic acids, could neither successfully dislodge nor dissolve the HCS sealers [15]. Therefore, the need for solvents for HCS materials still remains a clinically relevant topic.

Citric acid is a colorless organic acid commonly tested in endodontic research for different purposes [16]. However, little attention has been paid to citric acid as a potential solvent for HCS sealers. Based on the existing evidence-based literature on the chemistry of HCS cements, citric acid induces the gradual dissolution of calcium-based hydration products, eventually compromising the structural integrity of the material [17]. Therefore, it can be assumed that if the citric acid can dissolve the HCS materials, the solution could potentially be used as a solvent during endodontic retreatment when indicated. 

The number of commercially available HCS materials, launched as powder/liquid formulations or premixed and ready-to-use pastes, has increased significantly within the last few years [12]. Five types of HCS materials are available. The first type of material is based on Portland cement, contains a radio-pacifier and no additives, and is mixed with water. The second type has various additives (e.g., calcium oxide, calcium carbonate), whereas, in the third type, the water is replaced by alternative vehicles [18]. The fourth and fifth types of HCS materials are the most widespread in modern endodontics. Although both types are tri-calcium silicate-based, type four has to be hand-mixed with water, while type five is already premixed and ready to use [18]. BioRoot RCS sealer (BR; Septodont, Saint-Maur-des-Fosses, France) is a widely investigated fourth-type material, which is provided in powder (tri-calcium silicate, zirconium oxide, povidone) and liquid (water, calcium chloride, polycarboxylate) formulations. It should be manually mixed before use [11]. TotalFill BC Sealer (TF; FKG, La Chaux-de-Fonds, Switzerland) is one of the first introduced premixed formulations (fifth generation) composed of tri-and dicalcium silicate, calcium phosphate monobasic, zirconium oxide, tantalum oxide, calcium hydroxide, filler, and thickening agents [11]. Bio-C Sealer (BIOC; Angelus, Londrina, Brazil) is another premixed fifth-generation HCS sealer containing tri-and dicalcium silicate, tri-calcium aluminate, calcium oxide, zirconium oxide, silicon oxide, polyethene glycol, and iron oxide [12]. AH, Plus Bioceramic Sealer (AHPB; Dentsply Sirona, Ballaiques, Switzerland) is a relatively new also premixed fifth-type HCS material composed of zirconium dioxide, tri-calcium silicate, dimethyl sulfoxide, lithium carbonate, and thickening agents [12]. However, it should be highlighted that all these materials have the same biological properties and differ slightly in their chemical compositions and physical properties [10].

The solvent must have a disintegrational and deterioration effect on the appropriate material and simultaneously avoid exerting detrimental effects on the dentin microstructure. It has been demonstrated that scanning electron microscopy (SEM) is a precise method widely used in endodontic research to observe the topographic profiles of the surfaces of instruments, materials, or root dentine [19,20,21]. Since little is known about the interaction of citric acid with commonly used HCS sealers and root canal dentine, the present study aimed to assess the potential impact of 10% and 20% citric acid solutions on HCS materials and root canal dentine using SEM. The tested hypothesis was that citric acid possesses dissolving capability on HCS sealers with no adverse effect on root canal dentine.

## 2. Materials and Methods

### 2.1. HCS Sealers Preparation

Four HCS materials to be tested (AH Plus Bioceramic Sealer, Bio-C Sealer, BioRoot RCS and TotalFill BC Sealer) were prepared according to the manufacturer’s instructions. Plastic molds (5 mm diameter × 2 mm height) were used to standardize the samples. Molds filled with HCS sealers were incubated in culture tissue dishes sealed with polyethylene film at 37 °C. Samples were regularly sprayed with Hank’s balanced salt solution (HBSS) for the first 12 h (2 sprays of 0.1 mL per application every hour at the 20 cm source-to-object distance), aiming to provide moisture for the initial setting of sealers. Full immersion into storage solution within the first hours was avoided due to a high risk of material washout. After the initial setting, samples were stored in gelatinized HBSS for 59 days at 37 °C. At the end of incubation, the surfaces of all samples were polished with fine polishing discs. Samples were then gently removed from molds, rinsed in distilled water, and incubated in gas-permeable culture tissue dishes for 24 h at 37 °C to allow for liquid evaporation. Samples with cracks and pores detected on the radiographs were discarded. 

### 2.2. Visualization of HCS Exposure to EDTA and Citric Acid

Six samples of each completely set material were selected to visualize the interaction between HCS sealers and 17% EDTA (Cerkamed, Stalowa Wola, Poland), 10% citric acid, and 20% citric acid solutions. Both citric acid solutions were prepared by diluting 40% citric acid (Cerkamed, Stalowa Wola, Poland) with distilled water. Specimens were immersed in Eppendorf polypropylene tubes (Eppendorf, Hamburg, Germany) containing 2 mL of the tested irrigating solution. The experiment was repeated twice, and the visible outcome was recorded with a camera. 

### 2.3. Solubility of HCS Sealers

The minimum sample size was calculated using G*Power v.3.1 software (Heinrich Heine, Dusseldorf, Germany) with α error probability of 0.05 and 1-β error probability of 0.90. A required size of 8 specimens per group was determined. A total of 24 samples completely set of each material were weighed to an accuracy of 0.0001 g, and the mass was recorded as M_1_. Samples were then randomly allocated to three groups (n = 8): 17% EDTA (served as a control), 10% citric acid, and 20% citric acid. 

HCS samples were fully immersed in 2 mL of test solution for 5 min. Afterwards, samples were rinsed in distilled water for 1 min and incubated in gas-permeable culture tissue dishes for 24 h at 37 °C. The mass determined after the incubation was marked as M_2_. The percentage difference between the initial mass (M_1_) and final mass (M_2_) was calculated to express the material’s solubility in the immersion solution. 

### 2.4. Statistical Analysis

Statistical analysis was performed using RStudio software v.4.1.1 (RStudio Inc., Boston, MA, USA). The Shapiro–Wilk test, followed by the Levene’s test, was applied to determine the assumption of normality and the homogeneity of variance, respectively. One-way analysis of variance (ANOVA) along with Tukey’s test were selected to detect the statistically significant differences among groups at the confidence level of 0.95.

### 2.5. Root Canal Preparation

Nine intact and fully developed single-rooted lower premolars from patients aged 15–20 years were selected for this preliminary observation. Vital teeth were extracted for medical reasons and subsequently used in the study under the approval of the local ethics committee (protocol no. EK-2). Teeth were stored in 0.5% Chloramine-T trihydrate solution at 4 °C until the start of preparation. 

Conventional endodontic access cavities were prepared using size No.2 EndoAccess burs (Dentsply Sirona, Ballaigues, Switzerland). The root canal length was determined by inserting a size 10 K-file into the canal until the tip was visible at the apical foramin, and the working length (WL) was established 1 mm short of the root canal length. Teeth were then randomly allocated to experimental groups (n = 3) depending on the irrigating solution applied during the preparation of root canals: 17% EDTA, 10% citric acid, or 20% citric acid. 

The glide path was created using size 15 and 20 K-Flexofile (Dentsply Sirona, Ballaigues, Switzerland) instruments. Further root canal shaping was performed with Hylex EDM instruments (Coltene-Whaledent, Allstetten, Switzerland) to the full WL in the following sequence: 25/0.12 Orifice opener, 25/~HyFlex OneFile, 40/0.04 Finishing File, 50/0.03 Finishing File. EDM instruments, powered by CanalPro Cordless Handpiece (Coltene-Whaledent, Allstetten, Switzerland), were used at a rotation speed of 500 rpm and a torque control level of 2.5 N/cm according to the manufacturer’s recommendations. During the shaping, root canals were repeatedly irrigated with 2 mL of appropriate irrigating solution for 2 min after each instrument change. As a final flush, 5 mL of sterile distilled water was used. The 5 mL syringes and 31-G NaviTip irrigation needles (Ultradent Products Inc., South Jordan, UT, USA) were used to deliver the irrigating solutions. All root canal shaping and cleaning procedures were performed by a single operator, an experienced endodontist.

After the preparation, root canals were dried with paper points. Teeth were carefully grooved longitudinally on the buccal and lingual surfaces without penetrating the root canal space. The specimens were then gently split into half and subjected to observation under a scanning electron microscope (SEM).

### 2.6. Scanning Electron Microscopy

The surface morphology of HCS sealers was evaluated using SEM Hitachi TM3000 (Hitachi, Tokyo, Japan), with an accelerating voltage of 15 kV. Three random specimens from each test group were attached to an aluminum stub and examined at ×1.0 k magnification. 

Analysis of the dentin surface was performed using SEM Hitachi SU-70 (Hitachi, Tokyo, Japan). Specimens were dried in a vacuum desiccator, coated with silver, and examined at ×500, ×2.5 k, and ×5.0 k magnifications with an accelerating voltage of 2 kV. 

## 3. Results

### 3.1. Visualization of HCS Exposure to EDTA and Citric Acid

**Figure 1:**
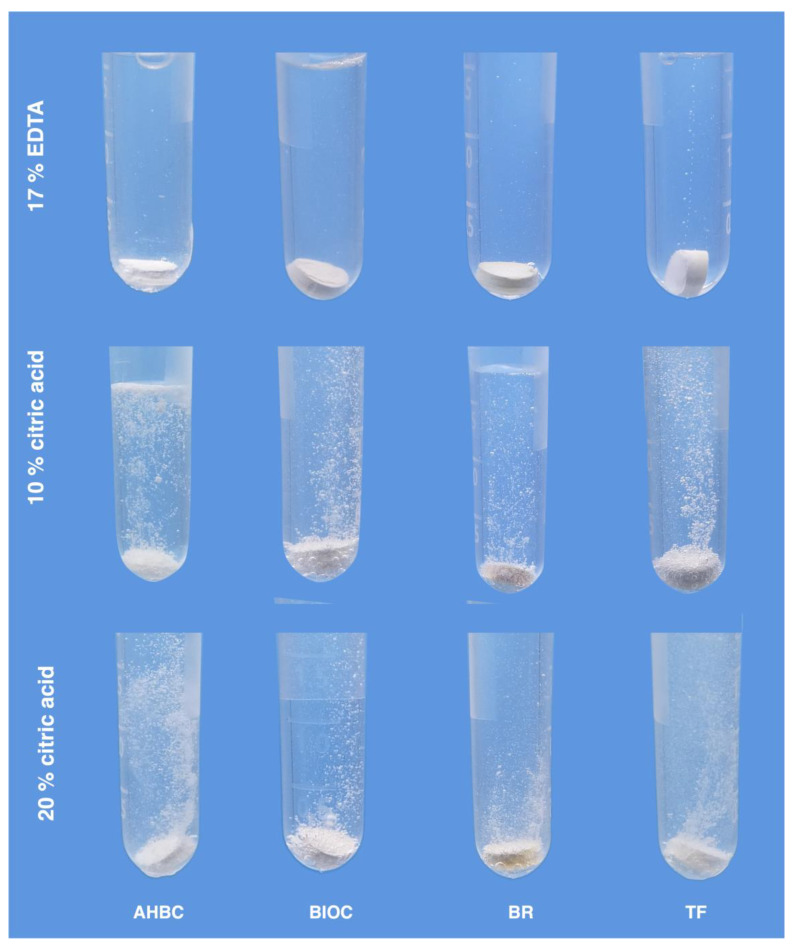
Representative images of HCS materials submerged into organic acids and demonstrating different intensities of “bubbling”.

Representative images visualizing the exposure of HCS sealers to 17% EDTA and 10% and 20% citric acid solution are shown in Figure 1. The more intense dissolution-related “bubbling” was visible in both citric acid solutions compared to EDTA. Meanwhile, the intensity of the gas release in 10% and 20% citric acid solutions was visually similar. The bubbling continued for all tested 5 min periods.

Considering the differences among the HCS sealers tested, all filling materials submerged in 17% EDTA revealed similar behavior, associated with the formation of only a few gas bubbles. On the contrary, the “bubbling” seen in citric acid solutions was apparently different, with the AHPB sealer demonstrating the most intense gas release. 

### 3.2. Solubility of HCS Sealers

The solubility of HCS sealers, to a certain degree, was determined for all tested solutions (Table 1). The highest solubility of all pre-mixed filling materials (AHPB, BIOC, and TF) was observed in 20% citric acid, followed by 10% citric acid. Meanwhile, BR demonstrated controversial results, being the most soluble in 10% citric acid. However, no tested HCS sealers showed statistically significant differences in mass loss between the 10% and 20% citric acid solutions. Further, both citric acid solutions were significantly more effective solvents for HCS materials compared to 17% EDTA.

### 3.3. Surface Characterization of HCS Sealers

**Figure 2:**
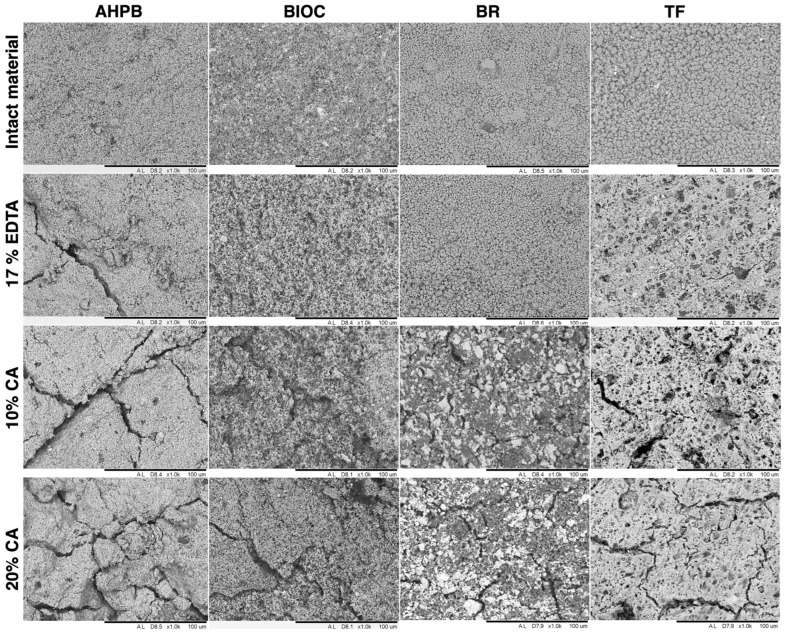
Representative SEM images of tested HCS materials before and after exposure to EDTA and citric acid (CA) solutions (×1.0 k magnification).

The superior dissolving properties of the 10% and 20% citric acid solutions were equally confirmed by SEM analysis of the treated HCS sealers (Figure 2). Morphological changes associated with pores and crack formation were clearly visible for all HCS materials exposed to citric acid solutions. Meanwhile, the damage to structural integrity provoked by EDTA was apparently less pronounced, and was mainly noticed for the AHPB sealer. 

### 3.4. Surface Characterization of Root Dentin

**Figure 3:**
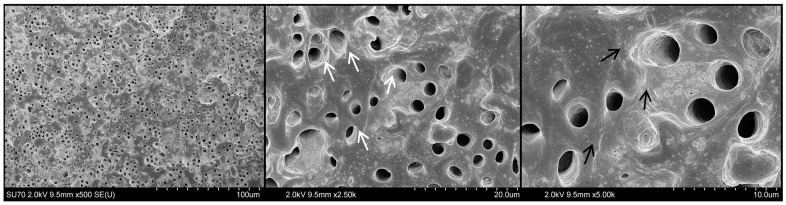
Representative SEM images of dentin surfaces conditioned with 17% EDTA. Magnifications at ×500, ×2.5 k, and ×5.0 k (**left** to **right**). White arrows indicate some widened and eroded dentinal tubules. Black arrows show areas of exposition of collagen fibrils.

**Figure 4:**
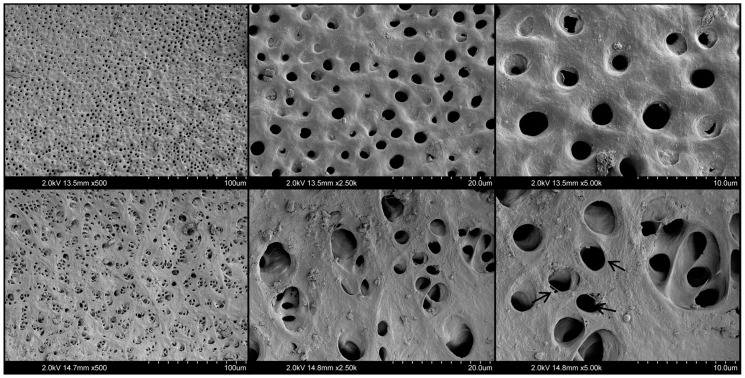
Representative SEM images of dentin surfaces conditioned with 10% citric acid. Magnifications set at ×500, ×2.5 k, and ×5.0 k (**left** to **right**). Arrows show areas of exposed collagen fibrils.

**Figure 5:**
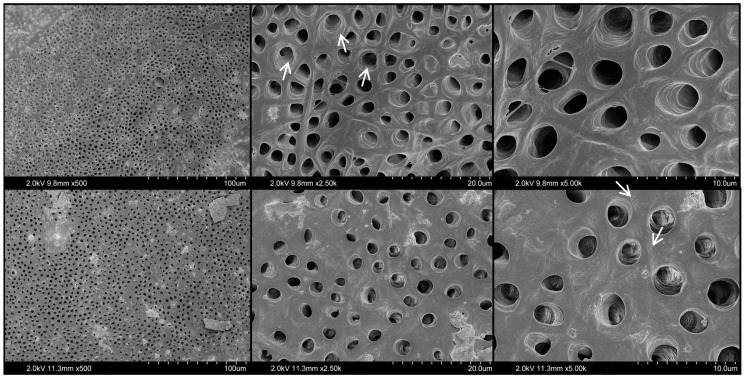
Representative SEM images of dentin surfaces conditioned with 20% citric acid. Magnifications at ×500, ×2.5 k, and ×5.0 k (**left** to **right**). Arrows indicate some widened and eroded dentinal tubules.

Representative images of the root canal segments treated with 17% EDTA, 10% citric acid, and 20% citric acid solutions are shown in Figure 3, Figure 4 and Figure 5, respectively. The EDTA mainly resulted in widening of the dentinal tubules, with some erosion and exposition of collagen fibrils. No evident structural changes were observed for 10% citric acid-treated dentine, demonstrating relatively regular and smooth outlines of most dentinal tubules. Meanwhile, the 20% citric acid resulted in relatively minimal peritubular and intertubular erosion, associated with rough and wider orifices of some dentin tubules. However, none of the tested groups demonstrated severe damage to dentine integrity nor to surface structure.

## 4. Discussion

This initial observation evaluated the dissolving potential of different concentrations of citric acid solutions on the fourth and fifth types of HCS sealers. Also, the potential detrimental impact of these irrigants on the microstructure of root dentine was examined. The findings indicated that citric acid at different concentrations could effectively dissolve all tested HCS materials without causing adverse effects on the root canal dentine. Therefore, the null hypothesis was accepted.

It has been shown that using different organic solvents or heat facilitates the removal of gutta-percha during endodontic retreatment [7]. However, the removal of root canal sealers, particularly based on HCS, is considerably more challenging owing to the higher physicochemical stability of these materials and, thus, the increased resistance to different solvents [6,15]. Currently, it is generally assumed that no specific and compelling solvent exists for HCS sealers [22]. Therefore, the mechanical removal of the previously placed HCS filling material remains the only clinically available option for endodontic retreatment procedures.

Published studies demonstrate that the retreatability of root canals filled with HCS sealers is possible, and that root canal patency can be regained in 91.67–100% of cases, depending on the tooth type [23,24]. However, the retreatment procedure is more challenging and time-consuming compared to some sealers with different chemical compositions [24]. It has been shown that removal of the HCS materials requires twice as much time as the resin-based or zinc oxide eugenol-based sealers [24]. Additionally, removing HCS sealers from larger root canals requires even more time, as the higher quantity of the sealer must be removed mechanically [23]. It should also be highlighted that internal root canal anatomy is usually very complex, and endodontic instruments cannot ensure complete root canal debridement and filling material removal [25,26]. Aiming to touch a higher surface area of the root canal walls, instruments bigger in size and taper might be used [25]. However, this generally results in excessive dentin removal, thereby weakening the root and increasing the risk of fracture. Therefore, a solvent capable of dissolving HCS materials would be a highly desirable clinical means to facilitate all these endodontic retreatment procedures [8,15].

Previously, M. Garrib and J. Camilleri (2020) [15] demonstrated that 10% formic acid and 17% EDTA might be efficient solvents, which, in conjunction with mechanical instrumentation, achieve over 95% removal of gutta-percha and HCS sealer [15]. However, other studies have not revealed any significant dissolving effect of irrigating solutions on HCS materials [8,27]. Surprisingly, citric acid as a potential solvent has not been tested until now, and there are no data available on the efficiency of citric acid for the dissolution of HCS sealers. Therefore, our preliminary findings, demonstrating the promising characteristics of 10% and 20% citric acid solutions, cannot be compared to previous investigations.

The hypothesis to test citric acid on HCS sealers mainly arose from the fact that the hydration products of di- and tri-calcium silicates become unstable and degradable at a pH below 8.8 [17]. Since the pH of citric acid solutions is typically below 2, it may explain the superior dissolving properties of citric acid compared to EDTA, which provides more alkaline conditions with a pH range from 7.5 to 9.5 [16]. Moreover, it should also be mentioned that in our study, the efficiency of citric acid solutions was clearly visible for both types of HCS sealers (mixed by hand or premixed), as dissolution and structural damage were objectively observed for all tested HCS materials, which were distinct in their chemical compositions and preparations.

The solvent’s interaction with root canal dentin during endodontic retreatment procedures is also inevitable. Generally, the potential adverse effect of the irrigating solutions on the root dentin microstructure directly relates to the concentration and exposure time [16]. Regarding the longer time required for HCS sealers to be removed from root canals compared to other types of sealers, prolonged exposure of the solvent to root dentin should always be expected [23]. Therefore, aiming to mimic the clinical situation and simulate the possible time-dependent effect of citric acid on the root dentine, prolonged exposure time was applied in this preliminary observation by using repetitive citric acid irrigation after each instrument. During the root canal shaping procedures, no use of NaOCl was included, exceptionally evaluating only the citric acid’s effect on the root dentin. The obtained results of SEM observations revealed that 10% and 20% citric acid solutions, even after a relatively long exposure, have no significant impact on the microstructure of root dentin. The exposed collagen fibers were observed only for EDTA-treated dentin in some areas. However, no significant erosive damage was noticed. These results agree with previous data demonstrating that EDTA alone does not adversely affect the dentin microstructure and that only additional subsequent application of NaOCl is related to erosion on the surface of the root dentine [16,28].

The interaction of HCS sealers and their behavior in citric acid solutions has not been investigated and visualized previously. Depending on HCS sealers’ chemistry and the hypothetical assumption that these materials in contact with chelating solutions can start to degrade, the tested HCS sealers were submerged in 17% EDTA and 10% and 20% citric acid solutions. The intense gas release and material physical changes reflected the ongoing chemical reaction between the HCS material and citric acid solutions at both concentrations. On the contrary, no evident bubbling was seen in the EDTA solution, indicating the absence of an intense chemical reaction. These observations support the previous findings demonstrating that HCS materials have very minimal solubility in EDTA [15]. Therefore, this irrigating solution has no evident potential to be used as a solvent for HCS sealers during endodontic retreatment procedures.

Additionally, it should be highlighted that the intensity of gas release visually differed among the materials tested, and was apparently most intensive for AHPB. The different degree of solubility was equally confirmed by the assessment of mass changes and surface morphology, demonstrating the highest mass loss and structural damage for AHPB, followed by BIOC, TF, and BR, respectively. The different acid resistance might be related to differences in the chemical composition and preparation of these materials. However, all these differences did not change the fact that all tested HCS sealers were significantly more soluble in citric acid solutions than EDTA. Therefore, our primary results suggest 10% and 20% citric acid solutions as potential solvents for HCS sealers, which could facilitate endodontic retreatment procedures. Further comprehensive investigations must be conducted to confirm or deny these results and, potentially, to modify the current clinical retreatment approaches for teeth previously filled with HCS material.

## Figures and Tables

**Table 1:** Mean and minimum–maximum percentages (%) of material mass loss in each immersion solution tested.

Sealer	17% EDTA	10% Citric Acid	20% Citric Acid
AHBC	14.19 (13.95–14.37) ^A^	22.27 (21.45–23.75) ^B^	23.42 (22.36–24.95) ^B^
BIOC	7.11 (6.48–8.42) ^C^	12.58 (11.08–15.01) ^D^	13.62 (12.02–15.63) ^D^
BR	3.05 (2.68–3.35) ^E^	6.51 (5.98–7.17) ^F^	6.29 (5.92–6.72) ^F^
TF	4.32 (3.92–4.81) ^G^	7.08 (6.61–7.42) ^H^	7.72 (6.91–8.24) ^H^

Values indexed with the same superscript letter in a row had no statistically significant differences (*p* > 0.05).

## Data Availability

Data are contained within the article.
